# Supplementary material for: Modelling reveals the effect of climate and land use change on Madagascar’s chameleons fauna
Source: Commun Biol. 2024 Jul 21;7:889. doi: 10.1038/s42003-024-06597-5 (PMC11271463; doi:10.1038/s42003-024-06597-5)
Supplement: Supplementary file 2 — Supplementary Information [file 42003_2024_6597_MOESM2_ESM.pdf]

Supplementary Information

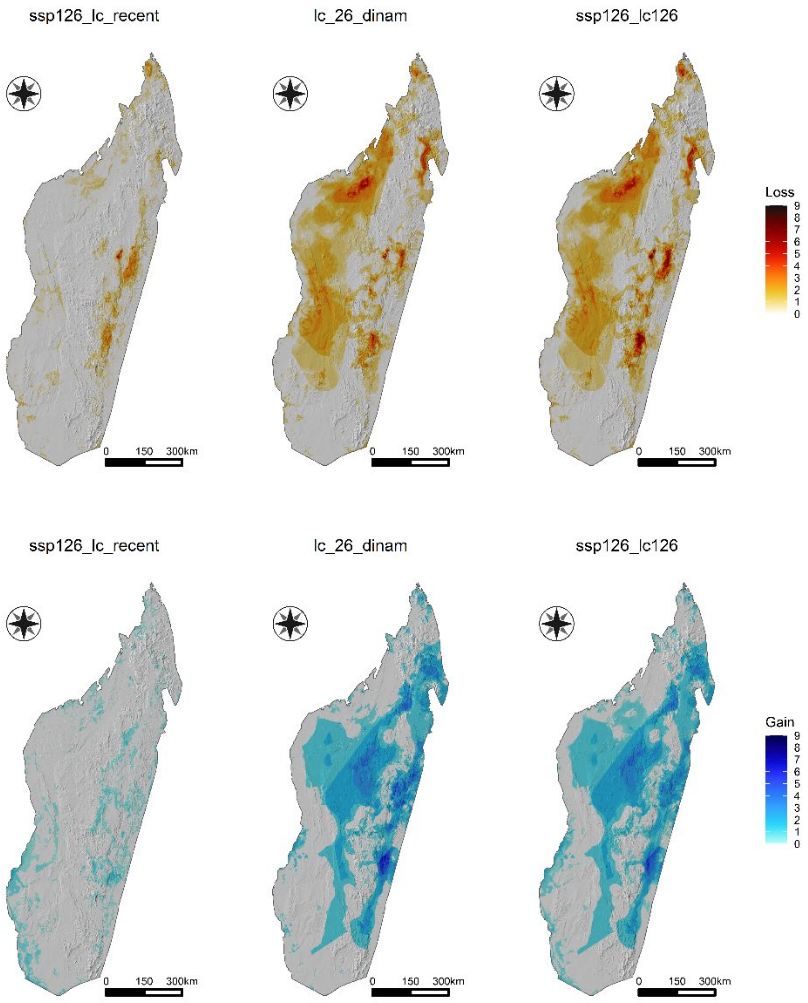

Supplementary Figure 1 = Species loss and gain of the Chamaeleonidae species future projected distributions under the mild SSP scenario considering the dynamic climate ("ssp126_lc_recent"), dynamic land use ("lc_26_dinam"), and the dynamic land-climate ("ssp126_lc26"). Predictions were obtained by averaging the results derived from all thresholds and GCM combinations.

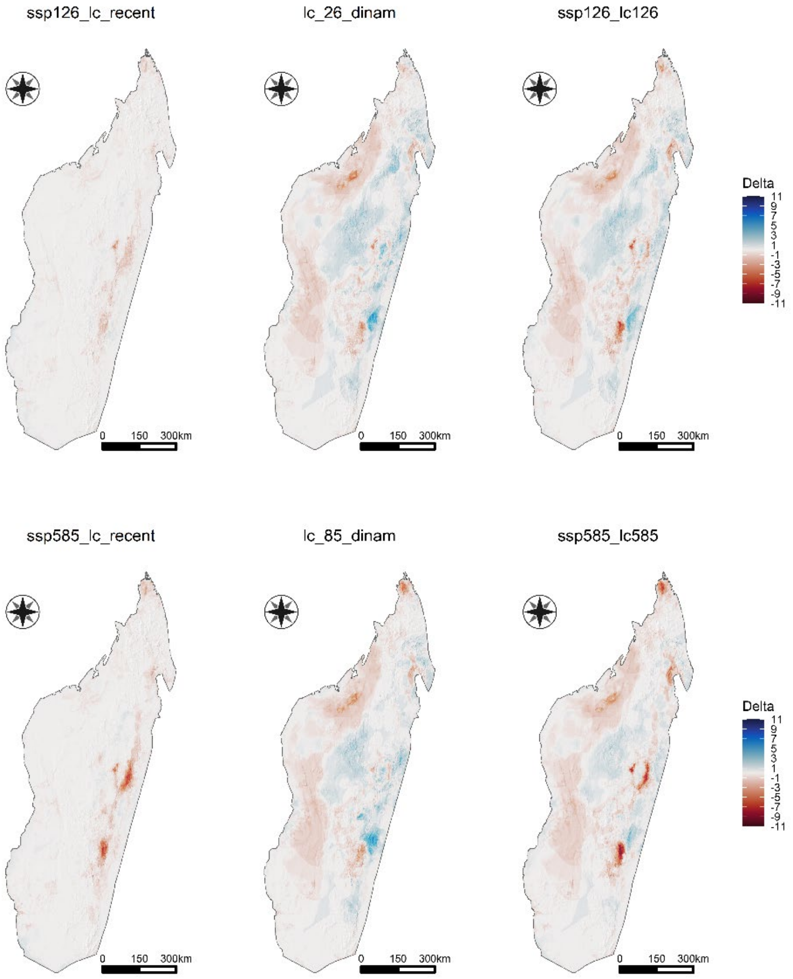

Supplementary Figure 2 = Change in richness of the Chamaeleonidae species future projected distribution under the mild and severe SSP scenarios. Predictions were obtained by averaging the results derived from all thresholds and GCM combinations. Colour bar indicates area with descrease (red) and increase (blue) in species richness comparing the current and all the future scenarios.
