# Supplementary material for: Modelling reveals the effect of climate and land use change on Madagascar’s chameleons fauna
Source: Commun Biol. 2024 Jul 21;7:889. doi: 10.1038/s42003-024-06597-5 (PMC11271463; doi:10.1038/s42003-024-06597-5)
Supplement: Supplementary file 3 — Description of Additional Supplementary Files [file 42003_2024_6597_MOESM3_ESM.pdf]

Description of Additional Supplementary Files

File name: Supplementary Data 1
Description: The chameleon tree in Newick format.

File name: Supplementary Data 2
Description: Raw occurrence data.

File name: Supplementary Data 3
Description: Species gain and loss in terms of geographic range under each model scenario

File name: Supplementary Data 4
Description: Species per ecoregion

File name: Supplementary Data 5

Description: R scripts
